# Utilization of Orthobiologics by Sports Medicine Physicians: A Survey-based Study

**DOI:** 10.5435/JAAOSGlobal-D-20-00185

**Published:** 2021-01-06

**Authors:** Peter C. Noback, Claire A. Donnelley, Nicholas C. Yeatts, Robert L. Parisien, James E. Fleischli, Christopher S. Ahmad, Claude T. Moorman, David P. Trofa, Bryan M. Saltzman

**Affiliations:** From the Department of Orthopaedics, Columbia University Medical Center, New York, NY (Noback, Donnelley, Ahmad, and Trofa); OrthoCarolina Sports Medicine Center (Yeatts, Fleischli, Moorman, and Saltzman); Atrium Health, Musculoskeletal Institute (Yeatts, Fleischli, Moorman, and Saltzman), Charlotte, NC; and the Department of Orthopaedics, University of Pennsylvania, Philadelphia, PA (Parisien).

## Abstract

**Introduction:**

Investigations are rapidly increasing into products referred to as orthobiologics and their utility in the nonsurgical and surgical treatment of diverse orthopaedic pathology.

**Methods:**

Members (599) of the American Orthopaedic Society for Sports Medicine were sent a survey that assessed their usage, motivation for use, and perceived efficacy of the following orthobiologics: leukocyte-rich platelet-rich plasma, leukocyte-poor platelet-rich plasma (PRP-LP), bone marrow aspirate concentrate, amniotic membrane products, adipose-derived mesenchymal stromal cells, and umbilical cord–derived cells. Application of these orthobiologics for the following pathologies was assessed: osteoarthritis, muscle injuries, tendon injuries, ligament injuries, labral injuries, and focal articular cartilage tears.

**Results:**

The survey was completed by 165 respondents (27.5%), of which 66.1% reported using at least one orthobiologic in their practice. Orthobiologic users reported the following: 71.6% are increasing their use, and 23.9% advertise their use. PRP-LP is the most commonly used orthobiologic for 76.1%, with 30% of PRP-LP users reporting use due to competitor utilization. The pathology most commonly treated with orthobiologics is osteoarthritis, for 71.6% of users, who primarily use PRP-LP in the knee joint. Leukocyte-rich platelet-rich plasma is the most popular orthobiologic in muscle, ligament, tendon, and labral injuries, whereas bone marrow aspirate concentrate is most popular for focal articular cartilage injuries. Primary orthobiologic-eligible groups were adults and recreational noncompetitive athletes. More than half (>50%) of orthobiologic users perceived all but umbilical cord–derived cells to be efficacious.

**Conclusion:**

Orthobiologics are used by a significant number of sports medicine physicians and are likely increasing in popularity. Among orthobiologics, platelet-rich plasmas are the most popular, and osteoarthritis is the pathology most likely to be treated. Orthobiologics are sometimes used for reasons other than clinical efficacy, especially competitor utilization, and physicians are disparate in their application of these products.

Rapidly advancing technology has driven awareness of the applicability of biologically derived materials to promote bone, ligament, muscle, and tendon healing in both professionals and patients.^1-3^ Broadly, these materials are referred to as orthobiologics. Concomitantly, research pertaining to the utility of these materials in the nonsurgical and surgical treatment of acute trauma, reconstructive procedures, and chronic degenerative pathologies has increased over the past decade.^4-6^ However, the exact formulation of each orthobiologic, the possible conditions for which they show promise, and the setting of their optimal application share one commonality: uncertainty.^7^ Furthermore, there is no agreed upon definition of what materials fall under the term orthobiologics, and the literature pertaining to optimal pathologies and settings for use is at best conflicting and at worst absent.^8^

When the medical literature conflicts, it is often left to the clinical judgment of physicians to determine the most appropriate treatment course for their patient. The diversity of treatment approaches is compounded by the quality of life focused nature of many orthopaedic interventions, orthobiologics in particular, which may expose surgeons to outside influences. One possible influence involves both physician and patient awareness of competitor's usage of these emerging technologies.^9^ Monetary incentives may also play a role.^10^ Despite these influences and the absence of universally adopted orthobiologic treatment guidelines, the extant literature is missing any assessment of orthopaedic surgeons' overall adoption of orthobiologics and motivations behind utilization.^6^ Because sports medicine–related knee, shoulder, and elbow-based complications specifically are often targets for adopting orthobiologic, injection-based therapies, how sports medicine physicians incorporate these treatments within their practices is of particular interest.

The purpose of this study was to assess the overall prevalence of orthobiologic usage within a representative group of high-level orthopaedic sports medicine practitioners. Additional study objectives included assessing surgeon usage, motivations for usage, and opinions of relative efficacy for orthobiologic types intended for various pathologies applied to multiple settings. We hypothesized that most respondents would be using at least one kind of orthobiologic in their practice, that competitor usage of orthobiologics would be a motivator for use, and that most respondents would agree that orthobiologics had at least some degree of efficacy.

## Methods

Members of the American Orthopaedic Society for Sports Medicine (AOSSM) were contacted by e-mail in August 2019 to participate in this study, for a total of 599 potential participants. The e-mail contained an anonymous link to a 158-item survey. The survey began by assessing demographic variables, followed by general usage questions about the following orthobiologics, chosen due to the frequency they appear in the literature: leukocyte-rich platelet-rich plasma (PRP-LR), leukocyte-poor platelet-rich plasma (PRP-LP), bone marrow aspirate concentrate (BMAC), amniotic membrane products (AMP), adipose-derived mesenchymal stromal cells (Ad-MSC), and umbilical cord–derived cells (UCD). Respondents were then asked detailed questions regarding the manner in which they used orthobiologics with respect to frequency, setting, and population. The survey concluded with several questions pertaining to each respondent's attitude toward orthobiologic usage. An outline of the manner in which the survey was organized can be found in Table 1. Respondents were sent a reminder e-mail at 2 weeks and then monthly for a 5-month open survey period if they did not complete the survey. Descriptive statistics were calculated and reported. A chi-square test of association was used to determine any relationship between time in practice (more/less than 10 years) and usage of orthobiologics (yes/no). A *P* value of less than 0.05 was considered significant.

**Table 1:** Questionnaire Outline

All Respondents (n = 165)
Section 1: demographics (3 items)
Geography
Years in practice
Practice composition
Utilization of any orthobiologics

Orthobiologic Users (n = 109)	Nonorthobiologic Users (n = 56)
Section 2: general, orthobiologics (3 items)	Section 2: motivations (1 item)
Advertise use of orthobiologics	Reason for lack of use
Trend of frequency of orthobiologic usage	
Specific orthobiologics used	
Sections 3 to 9: specific, individual orthobiologics^a^ (21 items per section)	
Utilization: age group, athletic groups, setting, and frequency	
Nonsurgical setting: qualifying pathologies for use and qualifying anatomic location	
Surgical setting: qualifying pathologies for use and qualifying anatomic location	
Efficacy of orthobiologic in practice	
Motives for use	

a Sections 3 to 9 each contained questions specific to one orthobiologic, which included leukocyte-rich platelet-rich plasma, leukocyte-poor platelet-rich plasma, bone marrow aspirate concentrate, amniotic membrane products, adipose-derived mesenchymal stromal cells, umbilical cord–derived cells, and other.

## Results

### Background Information

In total, 180 AOSSM members responded to the survey. Fifteen incomplete surveys were excluded, for a total of 165 respondents (complete response rate: 27.5%) who completed the survey in its entirety and were included in this analysis. Overall, 42 respondents (25.5%) practiced geographically in the East, 40 (24.2%) in the South, 50 (30.3%) in the Midwest, and 33 (20.0%) in the West. When asked to denote their practice structure, 125 respondents (75.8%) reported surgical sports medicine, 9 (5.5%) reported surgical shoulder/elbow, 24 (14.6%) reported general orthopaedic surgery, and 7 (4.2%) reported nonsurgical sports medicine. Of all respondents, 109 (66.1%) reported using at least one type of orthobiologic in their practice, with the same number (66.1%) of respondents reporting being in practice for over 10 years. No significant association was found between time in practice and orthobiologic usage (*P* = 0.324). Of the 109 respondents (66.1%) who use orthobiologics, 26 (23.9%) reported advertising their use of orthobiologics and 78 (71.6%) reported increasing their orthobiologic utilization.

**Figure 1:**
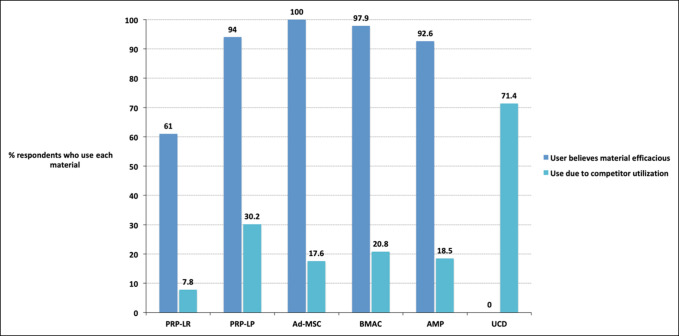
Clustered bar chart showing the percentage of users of each particular orthobiologic who believed that the product was efficacious when treating patients and percentage who attested to using a product because their competitors were using the product. Ad-MSC = adipose-derived mesenchymal stromal cells, AMP = amniotic membrane products, BMAC = bone marrow aspirate concentrate, PRP-LP = leukocyte-poor platelet-rich plasma, PRP-LR = leukocyte-rich platelet-rich plasma, UCD = umbilical cord–derived cell

The most common orthobiologic among the 109 respondents who incorporate at least one orthobiologic in their practice was PRP-LP, with 83 respondents (76.1%) reporting use. This was followed by PRP-LR for 77 (70.6%), BMAC for 48 (44.0%), AMPs for 27 (24.8%), Ad-MSC for 17 (15.6%), UCD for 7 (6.4%), and other for 2 (1.8%) (Figure 1). Unless otherwise indicated, the percentages of the remaining results are in reference to the 109 respondents who indicated that they use at least one orthobiologic.

### Orthobiologic-specific Responses

#### Platelet-Rich Plasma–leukocyte Rich

The percentages throughout the remainder of this paragraph are in reference to those 77 respondents who used PRP-LR. The most common patient age group for which PRP-LR was used was for adults, for 51 respondents (66.2%). For 4 respondents (5.2%), pediatric was least common. Within athlete type, college athletes were the most common, with PRP-LR use reported by 58 respondents (75.3%). The least common athlete type to receive PRP-LR were professional athletes for 33 respondents (42.9%). Fifteen respondents (19.5%) reported combining PRP-LR with hyaluronic acid. Forty-seven respondents (61.0%) agreed that PRP-LR demonstrated efficacy within their practice, and 6 (7.8%) agreed that they use PRP-LR because of competitor utilization.

#### Platelet-Rich Plasma–leukocyte Poor

The percentages throughout the remainder of this paragraph are in reference to those 83 respondents who used PRP-LP. The most common patient age group for which PRP-LRP was used was for adults, for 50 respondents (60.2%). For 0 respondents (0.0%), pediatric was least common. Recreational (noncompetitive) athletes were the most common athlete type for reported PRP-LP use for 51 respondents (61.4%). The least common athlete type to receive PRP-LP were college athletes for 27 respondents (32.5%). Seven respondents (8.4%) reported combining PRP-LP with hyaluronic acid. Seventy-eight respondents (94.0%) agreed that PRP-LP demonstrated efficacy within their practice, and 25 (30.2%) agreed that they use PRP-LP because of competitor utilization.

#### Adipose-derived Mesenchymal Stromal Cells

The percentages throughout the remainder of this paragraph are in reference to those 17 respondents who used Ad-MSC. The most common patient age group for which Ad-MSC was used was for adults, for 11 respondents (64.7%). For 0 respondents (0.0%), pediatric was least common. recreational (noncompetitive) athletes were the most common athlete type for reported Ad-MSC utilization for 11 respondents (64.7%). The least common athlete type to receive Ad-MSC were high school athletes for 0 respondents (0.0%). Two respondents (11.8%) reported combining Ad-MSC with hyaluronic acid. Seventeen respondents (100.0%) agreed that Ad-MSC demonstrated efficacy within their practice, and 3 (17.6%) agreed that they use Ad-MSC because of competitor utilization.

#### Bone Marrow Aspirate Concentrate

The percentages throughout the remainder of this paragraph are in reference to those 48 respondents who used BMAC. The most common patient age group for which BMAC was used was for adults, for 28 respondents (58.3%). For 2 respondents (4.2%), pediatric was least common. Recreational (noncompetitive) athletes were the most common athlete type for reported BMAC utilization for 32 respondents (66.7%). The least common athlete type to receive BMAC were high school athletes for 15 respondents (31.3%). Zero respondents (0.0%) reported combining BMAC with hyaluronic acid. Forty-six respondents (97.9%) agreed that BMAC demonstrated efficacy within their practice, and 10 (20.8%) agreed that they use BMAC because of competitor utilization.

#### Amniotic Membrane Products

The percentages throughout the remainder of this paragraph are in reference to those 27 respondents who used AMP. The most common patient age group for which AMP was used was for adults, for 17 respondents (63.0%). For 0 respondents (0.0%), pediatric was least common. Recreational (noncompetitive) athletes were the most common athlete type for reported AMP utilization for 22 respondents (81.5%). The least common athlete type to receive AMP were high school athletes for 7 respondents (25.9%). Four respondents (14.8%) reported combining AMP with hyaluronic acid. Twenty-five respondents (92.6%) agreed that AMP demonstrated efficacy within their practice, and 5 (18.5%) agreed that they use AMP because of competitor utilization.

#### Umbilical Cord–derived Cells

The percentages throughout the remainder of this paragraph are in reference to those seven respondents who used UCD. The most common patient age group for which UCD was used was for adults, for 3 (42.9%) respondents. For 0 respondents (0.0%), pediatric was least common. College athletes were the most common athlete type for reported UCD utilization for 7 respondents (100.0%). The least common athlete type to receive UCD were high school athletes for 1 respondent (14.3%). One respondent (14.3%) reported combining UCD with hyaluronic acid. Zero respondents (0.0%) agreed that UCD demonstrated efficacy within their practice, and 5 (71.4%) agreed that they use UCD because of competitor utilization.

### Pathologic-specific Responses and Utilization

**Figure 2:**
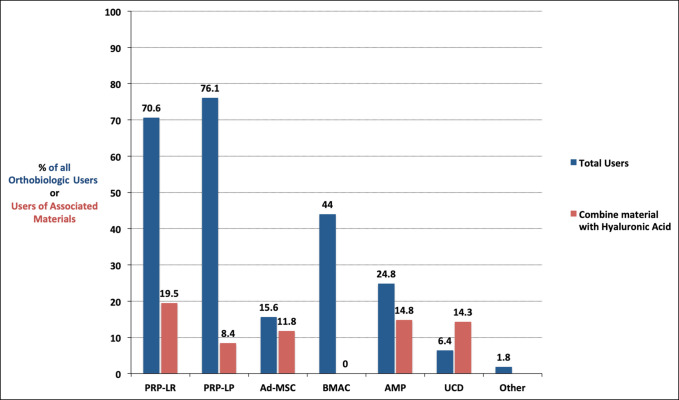
Clustered bar chart showing percentage utilization of each orthobiologic by respondents who used any orthobiologics and how often each orthobiologic was combined with hyaluronic acid by those who used each specific orthobiologic. Ad-MSC = adipose-derived mesenchymal stromal cells, AMP = amniotic membrane products, BMAC = bone marrow aspirate concentrate, PRP-LP = leukocyte-poor platelet-rich plasma, PRP-LR = leukocyte-rich platelet-rich plasma, UCD = umbilical cord–derived cell

Tables 2–7 contain results that detail orthobiologic usage by pathology and the most common location or pathology subset. These results are further stratified according to whether respondents reported orthobiologic use in a surgical or nonsurgical setting. Overall, the pathology for which an orthobiologic was most commonly used was osteoarthritis for 78 respondents (71.6%) of 109 orthobiologic users (Figure 2).

**Table 2:** Overall and Location-specific Usage of Orthobiologics in the Treatment of Osteoarthritis by Respondents

Osteoarthritis-specific Users	PRP-LR, 70.6% (n = 77)	PRP-LP, 76.1% (n = 83)	BMAC, 44.0% (n = 48)	AMP, 24.8% (n = 27)	Ad-MSC, 15.6% (n = 17)	UCD, 6.4% (n = 7)
Overall, 71.6% (n = 78)						
Nonsurgical, 56.9% (n = 62)	34.0% (n = 26)	62.7% (n = 52)	45.8% (n = 22)	66.7% (n = 18)	58.8% (n = 10)	57.1% (n = 4)
Nonsurgical location						
Shoulder	19.2%	50.0%	41.0%	55.6%	80.0%	75.0%
Elbow	11.5%	17.3%	9.1%	27.8%	20.0%	50.0%
Hip	19.2%	23.1%	36.4%	33.3%	100.0%	75.0%
Knee	92.3%	94.2%	91.0%	94.4%	40.0%	100.0%
Operative, 45.9% (n = 50)	20.8% (n = 16)	43.4% (n = 36)	39.6% (n = 19)	44.4% (n = 12)	58.8% (n = 10)	57.1% (n = 4)
Surgical location						
Shoulder	0%	33.3%	31.6%	41.7%	40.0%	50.0%
Elbow	0%	11.1%	12.5%	16.7%	10.0%	25.0%
Hip	6.3%	22.2%	31.6%	33.3%	10.0%	50.0%
Knee	93.7%	97.2%	94.7%	100.0%	100.0%	100.0%

Ad-MSC = adipose-derived mesenchymal stromal cells, AMP = amniotic membrane products, BMAC = bone marrow aspirate concentrate, PRP-LP = leukocyte-poor platelet-rich plasma, PRP-LR = leukocyte-rich platelet-rich plasma, UCD = umbilical cord–derived cells

Percentages in the top row and left-most column represent proportions with respect to all 109 users of any orthobiologics. Percentages throughout the rest of the table represent proportions with respect to the “n” value nearest vertically in the table. Specifically, percentages in the nonsurgical and surgical rows represent proportions with respect to all users of the orthobiologic indicated at the top of each column. Percentages in the rows below nonsurgical location and surgical location represent proportions with respect to all users of the column's specific orthobiologic in the indicated setting of nonsurgical or surgical.

**Table 3:** Overall and Location-specific Usage of Orthobiologics in the Treatment of Muscle Injuries

Muscle injury–specific Users	PRP-LR, 70.6% (n = 77)	PRP-LP, 76.1% (n = 83)	BMAC, 44.0% (n = 48)	AMP, 24.8% (n = 27)	Ad-MSC, 15.6% (n = 17)	UCD, 6.4% (n = 7)
Overall, 38.5% (n = 42)						
Nonsurgical, 27.5% (n = 30)	34.0% (n = 26)	14.5% (n = 12)	8.3% (n = 4)	7.4% (n = 2)	11.8% (n = 2)	14.3% (n = 1)
Nonsurgical location						
Biceps	19.2%	41.7%	50.0%	0.0%	100.0%	100.0%
Triceps	15.4%	33.3%	25.0%	0.0%	50.0%	0.0%
Gluteus	38.5%	41.7%	50.0%	50.0%	100.0%	0.0%
Hamstring	88.5%	91.7%	50.0%	50.0%	100.0%	0.0%
Surgical, 13.8% (n = 15)	11.7% (n = 9)	8.4% (n = 7)	6.3% (n = 3)	3.7% (n = 1)	17.6% (n = 3)	0.0% (n = 0)
Surgical location						
Biceps	33.3%	42.9%	66.7%	0.0%	66.7%	—
Triceps	22.2%	28.6%	33.3%	0.0%	33.3%	—
Gluteus	11.1%	42.9%	33.3%	0.0%	66.7%	—
Hamstring	88.9%	71.4%	66.7%	100.0%	66.7%	—

Ad-MSC = adipose-derived mesenchymal stromal cells, AMP = amniotic membrane products, BMAC = bone marrow aspirate concentrate, PRP-LP = leukocyte-poor platelet-rich plasma, PRP-LR = leukocyte-rich platelet-rich plasma, UCD = umbilical cord–derived cells

Percentages in the top row and left-most column represent proportions with respect to all 109 users of any orthobiologics. Percentages throughout the rest of the table represent proportions with respect to the “n” value nearest vertically in the table. Specifically, percentages in the nonsurgical and surgical rows represent proportions with respect to all users of the orthobiologic indicated at the top of each column. Percentages in the rows below nonsurgical location and surgical location represent proportions with respect to all users of the column's specific orthobiologic in the indicated setting of nonsurgical or surgical.

**Table 4:** Overall and Location-specific Usage of Orthobiologics in the Treatment of Tendon Injuries

Tendon Injury–specific Users	PRP-LR, 70.6% (n = 77)	PRP-LP, 76.1% (n = 83)	BMAC, 44.0% (n = 48)	AMP, 24.8% (n = 27)	Ad-MSC, 15.6% (n = 17)	UCD, 6.4% (n = 7)
Overall, 66.1% (n = 72)						
Nonsurgical, 66.1% (n = 72)	87.0% (n = 67)	41.0% (n = 34)	20.8% (n = 10)	40.7% (n = 11)	11.8% (n = 2)	28.6% (n = 2)
Nonsurgical location						
Rotator cuff	26.9%	35.3%	60.0%	72.7%	50.0%	50.0%
Triceps	11.9%	23.5%	20.0%	27.2%	50.0%	50.0%
Biceps	10.4%	32.4%	30.0%	27.2%	50.0%	50.0%
Flexor-pronator mass	47.8%	38.2%	20.0%	36.4%	100.0%	50.0%
ECRB	70.1%	32.5%	8.3%	54.5%	100.0%	100.0%
Quadriceps	32.8%	16.9%	30.0%	27.2%	100.0%	50.0%
Hamstring	29.9%	41.8%	60.0%	18.2%	100.0%	50.0%
Patella	73.1%	79.4%	40.0%	72.7%	100.0%	50.0%
Surgical, 42.2% (n = 46)	48.1% (n = 37)	24.1% (n = 20)	22.9% (n = 11)	22.2% (n = 6)	11.8% (n = 2)	0.0% (n = 0)
Surgical location						
Rotator cuff	48.6%	45.0%	72.7%	50.0%	50.0%	—
Triceps	10.8%	20.0%	27.3%	16.7%	50.0%	—
Biceps	10.8%	30.0%	18.2%	16.7%	50.0%	—
Flexor-pronator mass	29.7%	40.0%	27.3%	33.3%	100.0%	—
ECRB	54.1%	70.0%	45.5%	16.7%	100.0%	—
Quadriceps	24.3%	30.0%	45.5%	16.7%	100.0%	—
Hamstring	21.6%	35.0%	54.5%	16.7%	100.0%	—
Patella	59.5%	60.0%	54.5%	50.0%	100.0%	—

Ad-MSC = adipose-derived mesenchymal stromal cells, AMP = amniotic membrane products, BMAC = bone marrow aspirate concentrate, ECRB = extensor carpi radialis brevis, PRP-LP = leukocyte-poor platelet-rich plasma, PRP-LR = leukocyte-rich platelet-rich plasma, UCD = umbilical cord–derived cells

Percentages in the top row and left-most column represent proportions with respect to all 109 users of any orthobiologics. Percentages throughout the rest of the table represent proportions with respect to the “n” value nearest vertically in the table. Specifically, percentages in the nonsurgical and surgical rows represent proportions with respect to all users of the orthobiologic indicated at the top of each column. Percentages in the rows below nonsurgical location and surgical location represent proportions with respect to all users of the column's specific orthobiologic in the indicated setting of nonsurgical or surgical.

**Table 5:** Overall and Location-specific Usage of Orthobiologics in the Treatment of Ligament Injuries

Ligament Injury–specific Users	PRP-LR, 70.6% (n = 77)	PRP-LP, 76.1% (n = 83)	BMAC, 44.0% (n = 48)	AMP, 24.8% (n = 27)	Ad-MSC, 15.6% (n = 17)	UCD, 6.4% (n = 7)
Overall, 32.1% (n = 35)						
Nonsurgical, 28.4% (n = 31)	37.7% (n = 29)	14.5% (n = 12)	12.5% (n = 6)	18.5% (n = 5)	5.9% (n = 1)	0.0% (n = 0)
Nonsurgical location						
UCL elbow	79.3%	50.0%	16.7%	20.0%	0.0%	—
Extra-articular knee	48.3%	75.0%	66.7%	40.0%	100.0%	—
Intra-articular knee	3.4%	0%	50.0%	60.0%	0.0%	—
Surgical, 20.2% (n = 22)	20.8% (n = 16)	9.6% (n = 8)	18.8% (n = 9)	14.8% (n = 4)	11.8% (n = 2)	14.3% (n = 1)
Surgical location						
UCL elbow	43.8%	37.5%	22.2%	25.0%	0.0%	100.0%
Extra-articular knee	75.0%	62.5%	55.6%	25.0%	100.0%	0.0%
Intra-articular knee	25.0%	50.0%	66.7%	50.0%	0.0%	0.0%

Ad-MSC = adipose-derived mesenchymal stromal cells, AMP = amniotic membrane products, BMAC = bone marrow aspirate concentrate, PRP-LP = leukocyte-poor platelet-rich plasma, PRP-LR = leukocyte-rich platelet-rich plasma, UCD = umbilical cord–derived cells, UCL = ulnar collateral ligament

Percentages in the top row and left-most column represent proportions with respect to all 109 users of any orthobiologics. Percentages throughout the rest of the table represent proportions with respect to the “n” value nearest vertically in the table. Specifically, percentages in the nonsurgical and surgical rows represent proportions with respect to all users of the orthobiologic indicated at the top of each column. Percentages in the rows below nonsurgical location and surgical location represent proportions with respect to all users of the column's specific orthobiologic in the indicated setting of nonsurgical or surgical.

**Table 6:** Overall and Location-specific Usage of Orthobiologics in the Treatment of Labral Injuries

Labral Injury–specific Users	PRP-LR, 70.6% (n = 77)	PRP-LP, 76.1% (n = 83)	BMAC, 44.0% (n = 48)	AMP, 24.8% (n = 27)	Ad-MSC, 15.6% (n = 17)	UCD, 6.4% (n = 7)
Overall, 11.9% (n = 13)						
Nonsurgical, 8.3% (n = 9)	6.5% (n = 5)	6.0% (n = 5)	8.3% (n = 4)	7.4% (n = 2)	5.9% (n = 1)	0.0% (n = 0)
Nonsurgical location						
Hip	20.0%	20.0%	50.0%	100.0%	100.0%	—
Shoulder	80.0%	80.0%	100.0%	50.0%	100.0%	—
Surgical, 10.1% (n = 11)	6.5% (n = 5)	6.0% (n = 5)	8.3% (n = 4)	7.4% (n = 2)	5.9% (n = 1)	0.0% (n = 0)
Surgical location						
Hip	0%	0%	25.0%	50.0%	100.0%	—
Shoulder	100%	100%	100.0%	100.0%	100.0%	—

Ad-MSC = adipose-derived mesenchymal stromal cells, AMP = amniotic membrane products, BMAC = bone marrow aspirate concentrate, PRP-LP = leukocyte-poor platelet-rich plasma, PRP-LR = leukocyte-rich platelet-rich plasma, UCD = umbilical cord–derived cells

Percentages in the top row and left-most column represent proportions with respect to all 109 users of any orthobiologics. Percentages throughout the rest of the table represent proportions with respect to the “n” value nearest vertically in the table. Specifically, percentages in the nonsurgical and surgical rows represent proportions with respect to all users of the orthobiologic indicated at the top of each column. Percentages in the rows below nonsurgical location and surgical location represent proportions with respect to all users of the column's specific orthobiologic in the indicated setting of nonsurgical or surgical.

**Table 7:** Overall and Location-specific Usage of Orthobiologics in the Treatment of Focal Articular Cartilage Tears

Focal Articular Cartilage Tear–specific Users	PRP-LR, 70.6% (n = 77)	PRP-LP, 76.1% (n = 83)	BMAC, 44.0% (n = 48)	AMP, 24.8% (n = 27)	Ad-MSC, 15.6% (n = 17)	UCD, 6.4% (n = 7)
Overall, 41.3% (n = 45)						
Nonsurgical, 26.6% (n = 29)	10.4% (n = 8)	15.7% (n = 13)	31.3% (n = 15)	29.6% (n = 8)	11.8% (n = 2)	57.1% (n = 4)
Nonsurgical location						
Shoulder	25.0%	76.9%	33.3%	50.0%	50.0%	50.0%
Elbow	12.5%	38.5%	13.3%	37.5%	50.0%	25.0%
Hip	12.5%	46.2%	33.3%	50.0%	50.0%	50.0%
Knee	87.5%	7.7%	100.0%	100.0%	100.0%	100.0%
Surgical, 38.5% (n = 42)	18.2% (n = 14)	15.7% (n = 13)	47.9% (n = 23)	25.9% (n = 7)	11.8% (n = 2)	57.1% (n = 4)
Surgical location						
Shoulder	7.1%	69.2%	26.1%	42.9%	100.0%	25.0%
Elbow	7.1%	23.1%	8.7%	14.3%	50.0%	25.0%
Hip	14.2%	38.5%	17.4%	28.6%	50.0%	25.0%
Knee	100%	100%	100.0%	100.0%	100.0%	100.0%

Ad-MSC = adipose-derived mesenchymal stromal cells, AMP = amniotic membrane products, BMAC = bone marrow aspirate concentrate, PRP-LP = leukocyte-poor platelet-rich plasma, PRP-LR = leukocyte-rich platelet-rich plasma, UCD = umbilical cord–derived cells

Percentages in the top row and left-most column represent proportions with respect to all 109 users of any orthobiologics. Percentages throughout the rest of the table represent proportions with respect to the “n” value nearest vertically in the table. Specifically, percentages in the nonsurgical and surgical rows represent proportions with respect to all users of the orthobiologic indicated at the top of each column. Percentages in the rows below nonsurgical location and surgical location represent proportions with respect to all users of the column's specific orthobiologic in the indicated setting of nonsurgical or surgical.

#### Osteoarthritis

With respect to osteoarthritis, PRP-LP was the most commonly used orthobiologic in both surgical (36 respondents, 33.0%) and nonsurgical (52 respondents, 47.7%) settings (Table 2). Within the osteoarthritis group, PRP-LP was most commonly used to treat the knee joint in both surgical (35 respondents, 97.2%) and nonsurgical (49 respondents, 94.2%) settings.

#### Muscle Injuries

With respect to muscle injuries, PRP-LR was the most commonly used orthobiologic in both surgical (9 respondents, 8.3%) and nonsurgical (26 respondents, 23.9%) settings (Table 3). Within muscle injuries, PRP-LR was most commonly used for muscles in the posterior compartment of the thigh (hamstrings) in both surgical (8 respondents, 88.9%) and nonsurgical (23 respondents, 88.5%) settings.

#### Tendon Injuries

With respect to tendon injuries, PRP-LR was the most commonly used orthobiologic in both surgical (37 respondents, 34.0%) and nonsurgical (67 respondents, 61.5%) settings (Table 4). Within tendon injuries, PRP-LR was most commonly used for patellar tendon injuries in both surgical (27 respondents, 73.1%) and nonsurgical (40 respondents, 59.5%) settings.

#### Ligament Injuries

With respect to ligament injuries, PRP-LR was the most commonly used orthobiologic in both surgical (16 respondents, 14.7%) and nonsurgical (29 respondents, 26.6%) settings (Table 5). Within ligament injuries, PRP-LR was most commonly used for extra-articular knee ligaments in surgical settings (12 respondents, 75.0%) and the ulnar collateral ligament of the elbow in nonsurgical (23 respondents, 79.3%) settings.

#### Labral Injuries

With respect to labral injuries, PRP-LR and PRP-LP were tied for the most commonly used orthobiologic in both surgical (five respondents, 4.6%) and nonsurgical (five respondents, 4.6%) settings (Table 6). For orthobiologics in both surgical and nonsurgical settings, the shoulder joint was most commonly treated.

#### Focal Articular Cartilage Tears

With respect to focal articular cartilage tears, BMAC was the most commonly used orthobiologic in both surgical (23 respondents, 21.1%) and nonsurgical (15 respondents, 13.8%) settings (Table 7). Within focal articular cartilage tears, BMAC was primarily used for the knee in both surgical (23 respondents, 100.0%) and nonsurgical (15 respondents, 100.0%) settings.

## Discussion

This study is the first to assess the utilization of orthobiologics by orthopaedic sports medicine practitioners, the population of orthopaedic surgeons most likely to use these materials. The primary metric was orthopaedic surgeon's overall usage of orthobiologics. Secondary findings included patient populations, pathology subgroups, and usage trends. Orthobiologics are fast becoming commonplace in sports medicine clinics across the globe.^6^ This was supported by our study, which found that over 66% of surgeon respondents use at least one orthobiologic in their practice, and 71.3% current orthobiologic users anticipated increasing their usage in the immediate future. This study is likely representative of orthopaedic sports medicine practitioners, as most respondents have been in practice for over 10 years, describe themselves as surgical sports medicine physicians, and reside all across the United States. Orthobiologics were primarily used in the adult population, as opposed to elderly and pediatric patients. For every orthobiologic other than PRP-LR, recreational, noncompetitive athletes were most commonly treated with orthobiologics.

When efficacy reports conflict in orthopaedics, utilization of a single intervention or treatment strategy is uncommon. Conversely, when consensus exists regarding emerging treatments, variability of interventions is minimal.^11,12,13,14,15^ Orthobiologics fall into the latter category and continue to gain popularity despite the limited evidence in support for or against their use.^6^ Our findings demonstrate the immediate need for orthobiologic research—both to standardize evidence-based treatment guidelines and to understand surgeon motivations behind orthobiologic utilization. This study demonstrated a spectrum of clinical practice regarding orthobiologic choice; for the treatment of any one pathology, at least five different orthobiologics (of six possible) were used. Furthermore, although most orthobiologic users supported the statement that orthobiologics are efficacious, they also attested that there were often multiple factors influencing their decision making; the share that was affected by the influence of competitor utilization was never less than 7.8% (Figure 1). In fact, for the most commonly used product PRP-LP, roughly 30% of users attested to using PRP-LP due to competitor influence. In short, orthobiologics are popular among sports medicine physicians, and popular for reasons outside of stringent scientific evidence, such as competition and anecdotal evidence of efficacy.

Overwhelmingly, the two varieties of platelet-rich plasma—leukocyte rich and leukocyte poor—were the most popular option among respondents, with over 70% of orthobiologic users reporting use of at least one (Figure 2). The vast amount of literature demonstrating platelet-rich plasma as an efficacious treatment compared with other orthobiologics is likely a factor in this result.^15-17^ Encouragingly, for both PRP-LP and PRP-LR, most users agreed that they were efficacious. In addition, the minority of users report combining an orthobiologic with hyaluronic acid—at most, 19.5% of users of a particular orthobiologic will combine it with hyaluronic acid, as seen with PRP-LR.

**Figure 3:**
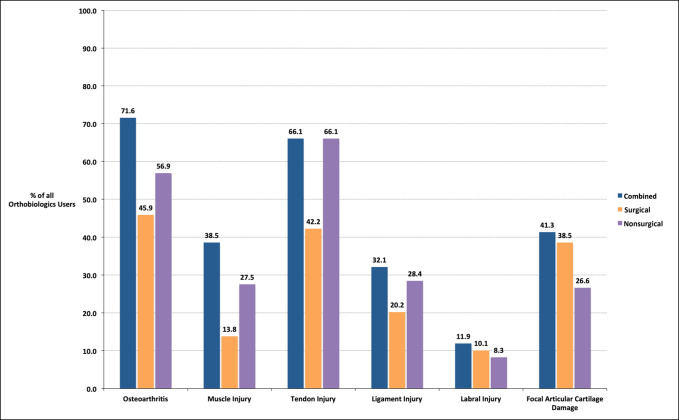
Clustered bar chart showing the percentage of orthobiologic users who applied at least one orthobiologic to the listed pathologies in any setting, the surgical setting, or the nonsurgical setting.

This study also examined orthobiologic usage by pathology. By far, the most commonly treated pathology was osteoarthritis, with 71.6% of orthobiologic users reporting applying orthobiologics to this pathology (Figure 3). The frequency that respondents attested to treating osteoarthritis with orthobiologics was also not accounted for by a select few materials. Although PRP-LP and PRP-LR were the most commonly used, every orthobiologic option surveyed was marked as being used in osteoarthritis by at least 3.7% of orthobiologic users. The high utilization of orthobiologics for osteoarthritis is consistent with the literature: among all surveyed pathologies, osteoarthritis likely has the most demonstrated efficacy, although there are exceptions.^18,19,20,21,22^ Coincidently and surprisingly, 23.9% of orthobiologic users reported using PRP-LR in the nonsurgical setting for osteoarthritis, despite substantial scientific evidence that the presence of leukocytes induces catabolic effects and an acute inflammatory response, which may actually prolong healing.^23,24,25,26^ The cause of this finding merits examination in future literature.

There are a number of limitations to our study. First, our response rate of complete surveys was 27.5%, and our study could thus be criticized as not providing a sufficient representation of our target population. However, the intention of our study was to obtain a high absolute number of responses from a representative pool of respondents (ie, members of the AOSSM representing all sports medicine surgeons across the country). For a global organization such as the AOSSM, membership is vast and complete return unlikely. With 165 complete surveys, the number of raw responses and distribution across the United States is reassuring that a sufficient, representative sampling was obtained. In addition, the number of total respondents assessed in our survey is similar in magnitude to many other survey-based studies in orthopaedics that assess treatment utilization.^27,28^ Second, survey components consisted of predetermined, rigid response choices, which could not reflect real-time clinical decision making. We considered a more free-form text entry survey to make results more representative of actual clinical practice, but such formatting would render results less concise and generalizable. Finally, respondents were not provided with clinical vignettes and were forced to make decisions based on simplistic scenarios. Thus, we could not capture how dogmatic surgeons are in their approach. Some surgeons may prefer a particular orthobiologic for one kind of patient and a completely different approach for another type. To maintain a thorough scope of topics (with 158 questions), we sacrificed free-text boxes to prevent an overly burdensome survey.

Orthobiologics are riddled with uncertainty. There is uncertainty in the orthobiologic definition, uncertainty in how orthobiologics should best be used, and uncertainty in the patient age, degree of physical fitness, and pathology most likely to benefit.^7^ The purpose of this study was to highlight the global adoption of orthobiologic use despite continued unknowns in their usage and efficacy, and the clear need for research to establish evidence-based practices around the utilization of these substances.

## Conclusion

Orthobiologics are used by a significant portion of surveyed sports medicine physicians, are increasing in popularity, and are largely thought of as efficacious, although they are sometimes used for reasons other than clinical efficacy. Platelet-rich plasmas are used most often, and osteoarthritis is the pathology most likely to be treated with an orthobiologic; however, physicians are far from streamlined in their application of these products. These findings illuminate orthobiologics as a rapidly expanding and divisive branch of orthopaedics that requires further research.
